# European Public Health News

**DOI:** 10.1093/eurpub/ckac137

**Published:** 2022-10-03

**Authors:** Marieke Verschuuren, Hans Henri P. Kluge, Gerald Rockenschaub, Hedinn Halldorsson, Daniela Martini, Kristina Ronsin, Oleg Storozhenko, Catherine A.H. Smallwood, Souaad Chemali, John Middleton, Floris Barnhoorn, Reinhard Busse

**Affiliations:** WHO Regional Office for Europe; WHO Regional Office for Europe; WHO Regional Office for Europe; WHO Regional Office for Europe; WHO Regional Office for Europe; WHO Regional Office for Europe; WHO Regional Office for Europe; Centre for International Health Protection, Robert Koch Institute, Berlin, Germany

In this edition of European Public Health News, we will reflect on past achievements and how we build on those for upcoming activities. In my first EUPHA office column I describe the busy yet exciting autumn the EUPHA team has ahead of them and highlight some of our main plans. Kluge *et al*. from the WHO Regional Office for Europe go into how lessons from COVID-19 and Ukraine are shaping the deployment and work of Emergency Medical Teams. Middleton reflects on ASPHER’s achievements related to ensuring a vibrant multidisciplinary public health workforce now that he is reaching the end of his term as President for that organization. Finally, Barnhoorn and Busse describe the plenary programme of the European Public Health Conference that will take place 9–12 November 2022 in Berlin. Topics range from financial protection and the European Health Data Space to health system performance assessment and health promotion.

## EUPHA office column: rolling up our sleeves for a lot of exciting work ahead

VerschuurenMarieke

This is the first office column I write as the new Executive Director of EUPHA. In July, I took over this function from Dineke Zeegers-Paget, who was in charge of the EUPHA office for 25 years. Under her guidance, EUPHA has grown tremendously as an organization, not only in size but also in scope and professionality. Obviously, I have very big shoes to fill! Luckily, I can count on the wonderful EUPHA office team to help and support me in this endeavour.

We have a busy autumn period ahead. A main milestone will be—hopefully—the first in person European Public Health (EPH) Conference since 2019. In-person meetings are picking up again, and it makes me realize how much I have missed genuine personal contact and chitchatting over coffee. Although working online gets the job done, physically meeting people is an indispensable element of true collaboration and building relationships. I am therefore very much looking forward to the EUPHA family coming together again during the EPH conference in Berlin.

In September, we will also start work on two new EU-funded projects: PROPHET and the European Health Data Space (EHDS2) pilot. PROPHET will develop a strategic research and innovation agenda for personalized Prevention. The EHDS2 pilot will develop an infrastructure for the future EHDS and test this through concrete research use cases. In both projects, EUPHA, in line with our science-based advocacy profile, will have a substantial role in stakeholder analysis and tailored dissemination of research outcomes. At the time of writing, we have posted a vacancy note for a project officer to support us in this work and strengthen our Brussels office.

Another major development for EUPHA is that we again obtained an operating grant (OG) from the European Commission to financially support us in the execution of our work plan for the period July 2022–June 2023. We see this as a recognition of the importance of our work for European public health and we greatly appreciate this opportunity. Among the activities that we will be able to strengthen due to the OG are supporting our members by means of capacity building activities and facilitating mutual learning, and working with our sections to make sure that we can provide the right evidence at the right time.

Under the previous EU Health Programme, OGs offered stable and predictable funding to NGOs, including EUPHA, enabling NGOs to actively participate in the health debate and make sure that the voice of civil society, including the most vulnerable, is heard.^1^ Although the importance of civil society engagement for ensuring transparent, fair and impactful policy measures never was clearer than during the pandemic, surprisingly, OGs originally were abolished in the current EU4Health Programme 2021–27. After concerted civil society action^2^, however, the financial mechanism was reinstalled under the 2022 work plan, albeit only for 1 year. Therefore, civil society, including EUPHA, is currently advocating a longer-term mechanism to be included in the 2023 work plan. This is a necessity as OGs are crucial for health NGOs to continue playing their essential role in EU policymaking processes and thereby key for safeguarding the democratic legitimacy of these processes.

## Lessons from COVID-19 and Ukraine: how ongoing health emergencies are shaping the deployment and work of Emergency Medical Teams

KlugeHans Henri P
WHO Regional Office for Europe
RockenschaubGerald
WHO Regional Office for Europe
HalldorssonHedinn
WHO Regional Office for Europe
MartiniDaniela
WHO Regional Office for Europe
RonsinKristina
WHO Regional Office for Europe
StorozhenkoOleg
WHO Regional Office for Europe
SmallwoodCatherine A H
WHO Regional Office for Europe
ChemaliSouaad
Centre for International Health Protection, Robert Koch Institute, Berlin, Germany

Following the 2010 earthquake in Haiti and the resulting lessons in strengthening disaster response and the provision of international support, the World Health Organization established the Emergency Medical Teams (EMTs) initiative to prepare for and promote a quality-assured, timely and coordinated response to rapid onset disasters.^1^

EMTs, in short, were set up to temporarily bridge gaps in the delivery of quality health services to affected populations in emergencies and provide support to national health systems. Each team consists of health professionals that have the necessary training, quality, equipment and supplies to swiftly and self-sufficiently respond to a crisis, without imposing a burden on the national health system. As of April 2022, around 100 teams were in the process of being internationally classified by WHO, while 32 teams already are—of which more than half are from the WHO European Region.^2^

Since the initiative was established, EMTs have been deployed to natural disasters, outbreaks and armed conflict, two examples being the 2014–15 Ebola Virus Disease and 2016–17 fighting in Mosul, Iraq.

In the WHO European Region, the COVID-19 pandemic and the war in Ukraine have clearly demonstrated the added value of EMTs as part of the global health workforce during emergencies.

COVID-19 provided an unconventional operational EMT context, particularly as the pandemic simultaneously challenged the resources of all countries. This was a health emergency that required extensive international support between European countries and led to repeated requests for EMTs, putting immense pressure on the initiative at a time when many EMTs were supporting domestic responses and therefore unable to respond to international requests. The pandemic response also required specific technical capacity within EMTs, including for infection prevention and control, and skills to manage severe acute respiratory infection cases. This is a different profile from the expertise, best practices and trainings required to address the health needs resulting from natural disasters or conflicts.

In response to the pandemic, EMTs were able to provide agile and hybrid support, travelling when needed but also supplementing with long-term remote support to countries. The response further revealed the importance of strengthening cross-collaboration and interoperability between EMTs and other existing rapid response capacities such as the Global Outbreak Alert and Response Network (GOARN), including Rapid Response Mobile Laboratories (RRMLs); jointly deployed in the context of complex emergencies.^3^^,^^4^

The war in Ukraine is another emergency that highlights the need for multilateral operation and coordination of EMTs.^5^ At the time of writing, three EMT Coordination Cells (EMTCCs) are active in Ukraine, the Republic of Moldova and Poland and have coordinated more than 60 organizations from all over the world since the start of the response. This intense cross-country operation requires thorough coordination with local authorities and stakeholders on the ground to ensure timely quality-care to populations in the most affected areas.

The requirements of responding to COVID-19 and the war in Ukraine, both large-scale, complex emergencies unfolding in a dynamic environment, have resulted in an evolution of the EMT Initiative. If applied broadly, more flexible deployments can extend the benefits of EMT standardization and quality assurance to multiple hazards and crises. Both responses highlight the need for increased coordination between health capacities. As an example, Public Health Rapid Response Teams and EMTs can easily develop better joint modalities for rapid response.

Moving forward and integrating lessons learned, EMTs should seek to build awareness about the Initiative and promote national EMT capacities. They should also develop and sustain a multi-functional EMTCC roster of surge capacities and establish systematic and informative EMT data management tools when needed. It doesn’t stop here. EMTs would also benefit from improving needs-assessment mechanisms that inform EMTCC decision-making, adopt a modularized approach and enhance national partnerships and opportunities for twinning between teams.

To take stock and map out the future direction in the light of ongoing responses, more than 500 participants from the EMT Network across WHO regions, comprised of teams, technical experts and international partners across organizations, will convene in Yerevan, Armenia, for the EMT 5th Global Meeting on 5–7 October 2022.^6^

To strengthen the governance of the EMT initiative, identify areas for future development of best practices and minimum standards, support capacity building at local, national and regional levels, and foster partnerships with other networks, the EMT’s 2030 strategy will be refined. Leveraging the benefits of standardization and flexibility, enhancing countries’ surge capacity targeting the rapid mobilization and coordination of national and international medical teams and the healthcare workforce, represent vital steps to address multi-hazard emergencies on a regular, consistent basis. A more modularized approach will also play an important role in an evolving future EMT mechanism.

Emergencies cannot be fully predicted. Yet one can prepare and plan for an efficient response that meets complex requirements, irrespective of where crisis hits. In that regard, COVID-19 and the war in Ukraine, offer unique learning opportunities that WHO intends to grasp in order to support Member States to prepare for and respond to future health threats—and to fully adapt the lessons of the past.

## Reflections on 3 years as President of the Association of Schools of Public Health in the European Region (ASPHER)

MiddletonJohn

I conclude my term of office as ASPHER President in October 2022. My term was extended for a year due to the extraordinary circumstances of the COVID-19 pandemic. I welcome the opportunity given me by EUPHA colleagues, to offer some reflections on what the public health community has achieved in the last 3 years, in this most challenging period.

### Introduction

ASPHER is the Association of Schools of Public Health in the European Region. ASPHER has over 130 members in 44 countries out of the 53 in the WHO European Region. Our core aim is improving and protecting the public health by strengthening education and training of public health professionals for both practice and research. When I took up office, my agenda was to deliver the core business of ASPHER, improve our governance, management, and partnerships. New aspects of our 2020–25 strategy were in building the young professional agenda and developing the corporate citizenship role of schools including in diversity and inclusion, social responsibility and carbon management.

Partnership working has been a defining aspect of most of my professional life so I will start with ASPHER’s commitment to partnership.

### European partnerships for health

We have collaborated strongly with EUPHA following our joint statement last year.^1^ We have had a strong presence at EPH online congresses and in European Public Health weeks. We joined with EUPHA in thanking the public health community for their work on COVID.^2^ We have moved forward work on public health ethics with EUPHA and the UK Faculty of Public Health. We continue to work strongly with the European Union. We have launched a statement on climate change and health education for the European Health Policy Platform, with more than 80 partner organisations.^3^ We have applied the Global Consortium on Climate Change and Health Education (GCCCHE) curriculum for climate change and health for a European audience.^4^ We have completed a commission for the European communicable Disease Surveillance Center (ECDC) on the revised core-competencies for applied infectious diseases epidemiology.^5^ We have also neared completion for ECDC on a project for vaccinology and vaccine hesitancy education (‘VXTrain’). ASPHER is an active member of the ECDC advisory board, one of three NGO members. We have also become a recognised Non-State Actor in the proceedings of WHO Euro. We have also reconvened the meeting of ASPHER, EPHA, EUPHA, EHMA and Eurohealthnet to share our strategic work, formulate joint policies and statements, recognize when there are opportunities for joint funding bids and to avoid wasteful competition for funding sources.

### Partnership working—with our global colleagues

ASPHER is a partner with the World Federation of Public Health Associations and the Italian Public Health Association (SITL) in next year’s World Congress on Public Health in Rome. We are major contributors to the work of the Global Network for Academic Public Health, with our sister associations in the WHO regions of the world. With GNAPH we have called for major investment in schools of public health.^6^ We must build the quality and quantity of training and capacity building for local national and international public health systems. ASPHER has contributed to a major WHO document, ‘National workforce capacity to implement the essential public health functions including a focus on emergency preparedness and response: roadmap for aligning WHO and partner contributions’.^7^ The report was launched during European Public Health week and will be used to inform the work of the International Negotiating Committee for the Pandemic Preparedness Treaty. We are also working closely with WHO European office on a range of projects including implementation of competencies^8^ and the professionalization road map.^9^

### Advocacy—for the health of the public

It is important that ASPHER does not duplicate on the advocacy roles of other European and global partners, so we will fall in behind them and lend our weight to their statements and policies where we can. These have been exceptional times though and ASPHER members have demanded our involvement in addressing the pandemic, and the war in Ukraine. Other areas where we lead advocacy are specifically in relation to education and training, as with climate change,^3^^,^^4^ and the diversity and inclusion.^10^

The ASPHER COVID task force marked the first two years of pandemic in March 2022, passing its 100th meeting in the process; members have contributed to over 100 papers on COVID-19 and its implications for society and health systems.^11^ Our task force on war has published a statement on the war in Ukraine^12^ and moved forward a programme of webinars, organized by the University of Bielefeld, on various aspects of war and conflict. We will continue to support colleagues working in Ukraine, and working on refugee health needs, where-ever they may be in Europe.

### The public health system, services and profession—ASPHER’s role supporting our members

We launched the WHO-ASPHER Competency Framework for the Public Health Workforce^8^ and the Roadmap for Professionalizing the Public Health Workforce. These are landmark building blocks for the public health community.^9^ They were developed by my predecessor Prof Kasia Czabanovska who now co-chairs the ASPHER-WHO professionalization task force. The road map provides tools for professionalization in public health. We need public health professionals who have been trained to a recognized set of competencies, and hold credentials which say they have achieved a high standard we need public health schools that operate to a core curriculum and which are preferably accredited to a high standard of education and teaching; we need public health systems which cover all the bases in health protection, health improvement and in health services evaluation and administration. We need the public to be protected from bad practice, through regulatory mechanisms and through the codes of conduct we use. And we need our professionals to be protected in their terms and conditions of their workplaces, and in the possibilities for career progression yet to be developed.^9^

We are revisiting the 5th edition of the ASPHER core-competencies document, under the leadership of Professor Mary Codd.^13^ We are moving forward other work on knowledge management. We have transferred ownership of the journal Public Health Reviews, to the Swiss School, but we continue to work closely with them and publish key statements in the journal. We are launching our new ASPHER website this month. Our involvement of young professionals continues and expands. Our new statutes give a place for an early career professional on the executive board, currently held by Ines Siepmann. We have a robust young professional steering group, and there is a budget for young professional related activity.

### ASPHER governance

We have continued to improve ASPHER’s governance—including implementation of new statutes from last year. Thirty new members have joined us in the last 3 years. Our finances have improved, and we have provided stronger support for members. We are seeking to further expand the secretariat and improve our office base in Brussels.

### Conclusion

The World is confronted with the COVID-19 pandemic and new infectious risks, armed organised violence, mass migration, extremes of heat and climatic conditions, appalling economic inequalities between rich and poor and between and within countries, and corporate over-exploitation creating addictions and cause non-communicable disease epidemics across the globe. We are challenged by the new information revolution which offers great opportunity to protect and improve the health of the public, but it also brings with it disinformation, surveillance of personal and beliefs and behaviours, and the undermining of economies and democracies we need as foundations for better health. All these threats require a stronger public health profession and stronger public health systems and services, from local to global. We need a vibrant multidisciplinary public health workforce, and we need new partnerships with disciplines with greater knowledge than ourselves- climate scientists, ecologists, economists, theologians, investigative journalists, political scientists and international lawyers.^14^ We need a united public health community if the health of people and the planet is to improve. ASPHER remains committed to working together with EUPHA and all our colleagues to ensure this vital work proceeds.

## 15th European Public Health Conference 9–12 November 2022 hub27 Berlin, Germany: strengthening health systems—improving population health and being prepared for the unexpected

BarnhoornFlorisBusseReinhard

**Opening session: strengthening health systems: improving population health and being prepared for the unexpected**

Wednesday 9 November 11:40 CET

Leaders from politics and academia will introduce the main theme of the conference. In the past years, we have learned from the COVID-19 pandemic that infectious diseases know no boundaries. Although we have seen the successful development of vaccines, we have also seen with the spread of the Delta and Omicron variants around the globe that these vaccines have not yet been available to everyone. At the conference, we want to explore ways in which the public health community can rally around the WHO health system framework to bring about better population health for all. The growing health inequalities around the globe lead to a simple conclusion: Europe must take on a more active role in global health. This includes taking a stand on issues such as climate change, environmental degradation, water and food security, migration, gender and poverty. Europe must use its knowledge and influence to balance out market forces, expand regulatory oversight and create socially responsible systems for health.

**Moderators:** Reinhard Busse, Chair of the 15th EPH Conference 2022; Verena Vogt, Chair of the International Scientific Committee

**Speakers:**

- Hans Kluge, WHO Regional Director for Europe
- Stella Kyriakides, Commissioner for Health and Food Safety, European Commission
- Karl Lauterbach, Minister of Health, Germany [tbc]

**Plenary 1: Can people afford to pay for health care? Evidence on inequity in financial protection in Europe**

Organizers: WHO Regional Office for Europe, Thursday 10 November, 14:50–15:50 CET

Governments have repeatedly affirmed their commitment to meeting the goals of universal health coverage (UHC)—to ensure that everyone can use the quality health services they need without experiencing financial hardship. Research from the WHO Regional Office for Europe shows that: gaps in health coverage lead to unmet need for health care and financial hardship among people using health services; these negative outcomes are heavily concentrated among people in poverty and those with multiple chronic conditions; countries can reduce unmet need and financial hardship by re-designing coverage policy. This session aims to raise awareness about the most prevalent gaps in coverage in European health systems, the policies that cause them and what countries can do to address them.

**Moderator**: Natasha Azzopardi Muscat, WHO Regional Office for Europe

**Keynote speaker:** Charles Normand, Trinity College Dublin, Ireland

**Panellists:**

- Sarah Thomson, WHO Regional Office for Europe
- Kaisa Immonen, European Patients’ Forum (EPF)
- Policy perspective from Germany (tbc)

**Plenary 2: Benefits and challenges of the European Health Data Space**

Organisers: EUPHA, European Commission, Thursday 10 November, 17:40–18:40 CET

The creation of a European Health Data Space (EHDS) is one of the key components of a strong European Health Union. The objectives of the EHDS are: (i) empower individuals through better digital access to their personal health data; (ii) fostering a genuine single market for digital health services and products; and (iii) set up strict rules for the use of individual’s non-identifiable health data for research, innovation, policy-making and regulatory activities. As such, the EHDS aims to improve and support healthcare delivery within Europe by allowing public health data to be accessible throughout Europe. The EHDS also aims to promote better access and exchange of different types of health data for research and policy purposes. This session will look into the challenges related to technology, governance and privacy in facing the creation of the EHDS.

**Moderators:** Iveta Nagyova, EUPHA; Isabel de la Mata, European Commission

**Speakers:**

- Andrzej Rys, European Commission
- Petronille Bogaert, Sciensano, Belgium, and president of EUPHA Public health monitoring and reporting section
- Irene Schlünder, TMF, Germany (tbc)
- Emmanuel Bacry, Health Data Hub, France (tbc)
- Representative of the European Patients’ Forum

**Plenary 3: Health Systems Performance Assessment for Policy: uses and abuses/measuring health system performance: using data to improve population health**

Organizers: European Observatory on Health Systems and Policies, European Commission

Friday 11 November, 10:30–11:30 CET

This plenary will look at the effective application of Health Systems Performance Assessment (HSPA) to health systems’ improvement as we face key challenges in the sustainability of our health systems. Too often the results of HSPA exercises, particularly when benchmarking is involved, are not well interpreted, and understood. When translating HSPA results into policy changes, we need to address a series of questions not only about the quality and validity of the indicators but, importantly, about the causal attribution and accountability implications and about the kinds of policy interventions required to address the performance failures.

**Moderators:** Isabel de la Mata, European Commission; Dimitra Panteli, European Observatory on Health Systems and Policies

**Keynote speaker:** Reinhard Busse, Technical University Berlin, Germany, and European Observatory on Health Systems and Policies

**Panellists:**

- Irini Papanicolas, London School of Economics, UK
- Jon Cylus, European Observatory on Health Systems and Policies

**Plenary 4: Reorienting health services: the transformational potential of health promotion**

Organizer: EuroHealthNet, Friday 11 November, 14:00–15:00 CET

This plenary session will provide examples of different ways in which health-promoting approaches can reorient health services, strengthen health-promoting and community oriented primary care and prevent chronic diseases. It will highlight what we can learn from behavioural and cultural insights and social prescribing, as well as integrated community initiatives to further support people, across the social gradient, to lead and to maintain healthy lives. It will discuss target setting for further advocacy among policy makers.

**Moderators:** Martin Dietrich, President EuroHealthNet, Caroline Costongs, Director EuroHealthNet

**Speakers:**

- Rüdiger Krech, World Health Organization
- Susan Michie, University College London, United Kingdom
- Jan De Maeseneer, Chair European Commission Expert Panel on Effective Ways of Investing in Health
- Jet Bussemaker, Council of Public Health & Society, Netherlands, Leiden University Medical Center, Leiden, Netherlands
- Cristiano Figueiredo, Central Lisbon Health Centre Cluster, National School of Public Health, NOVA University Lisbon, Portugal

**Plenary 5: Sustaining high-quality care: interprofessional training for our clinical and public health workforce**

Organized by ASPHER, EHMA, Saturday 12 November, 10:30–11:30 CET

**Moderators and Speakers:** tbc

**Closing session of the 15th EPH Conference**

Saturday 12 November, 15:00–15:45 CET

**Moderators:** Reinhard Busse, Chair of the 15th EPH Conference 2022; Verena Vogt, Chair of the International Scientific Committee

**Speakers:**

- Christian Drosten, Institute of Virology, Charité—Universitätsmedizin Berlin, Germany
- Dineke Zeegers Paget, past Executive Director EUPHA
- Anthony Staines, Chair of the 16th EPH Conference 2023 in Dublin

**Registration is still open, also onsite in Berlin**

More than 1500 colleagues have already registered for the biggest annual public health event in Europe. Attending the EPH Conference offers the following benefits: free access to over 175 parallel sessions, plenary sessions and lunch symposiums; Abstract Supplement of the *European Journal of Public Health*; Certificate of Attendance, Accreditation credits (UEMS).

Stay in touch: via our website—@EPHConference—#EPH2022
